# An Electronic Nose Based Method for the Discrimination of Weathered Petroleum-Derived Products

**DOI:** 10.3390/s18072180

**Published:** 2018-07-06

**Authors:** María José Aliaño-González, Marta Ferreiro-González, Gerardo F. Barbero, Jesús Ayuso, José A. Álvarez, Miguel Palma, Carmelo G. Barroso

**Affiliations:** 1 Department of Analytical Chemistry, Faculty of Sciences, IVAGRO, University of Cadiz, 11510 Puerto Real, Cadiz, Spain; mariajose.alianogonzalez@alum.uca.es (M.J.A.-G.); gerardo.fernandez@uca.es (G.F.B.); miguel.palma@uca.es (M.P.); carmelo.garcia@uca.es (C.G.B.); 2 Department of Physical Chemistry, Faculty of Sciences, Institute of Biomolecules (INBIO), University of Cadiz, 11510 Puerto Real, Cadiz, Spain; jesus.ayuso@uca.es (J.A.); joseangel.alvarez@uca.es (J.A.Á.)

**Keywords:** chemometrics, diesel, discrimination, eNose, environmental forensics, evaporation, fingerprints, gasoline, paraffin, petroleum-derived products, weathering

## Abstract

In recent years pollution due to hydrocarbon spills has increased markedly as a result of the numerous advances in technologies and industrial processes. Anthropogenic activities (accidental or illegal) are responsible for most of these incidents. In some cases, the spills are not detected at the moment they occur and the contaminants are subjected to different degradation phenomena that may change the chemical composition of the hydrocarbon over time. An incorrect or ineffective identification of the spill could lead to significant consequences, bearing in mind that most spills are hazardous to the environment. In the present work the capacity of the analytical technique based on the Electronic Nose (eNose) combined with chemometrics in the identification and discrimination of different weathered petroleum-derived products (PDPs) was studied. Different volumes (40 μL and 80 μL) of PDPs (gasoline, diesel, and paraffin) were poured onto different supports (wood, cork, paper, and cotton sheet) and subjected to a natural weathering process by evaporation for one month. The porosity of the support was also studied. The application of linear discriminant analysis allowed the full discrimination of the samples according to the presence/absence of PDP and a 97.7% of correct discrimination of the different PDPs regardless of the weathering time, support or volume used. The results show that the system is capable of detecting and discriminating the presence of petroleum-derived products in any of the situations studied.

## 1. Introduction

The rapid and dramatic growth in the population and technologies in recent years has had numerous consequences for the environment. The increase in the number of industries and the development of technologies and fuels are often accompanied by spills of petroleum-derived products (PDPs) with dangerous consequences for both nature and the nearby population [1,2]. These spills can arise due to human activities (accidental or illegal) or natural sources. For this reason, the identification of the spill is an important issue in environmental and forensic sciences. The main sources of anthropogenic operational discharges are related to production platforms, tanker vessels, municipal waste, and operational seepage. Such incidents are responsible for significant environmental damage. For this reason, the correct and rapid identification of the hydrocarbon source of a spill is a prerequisite to take appropriate action and minimize the hazards to the natural environment [3,4,5]. In addition, hydrocarbons such as gasoline, diesel or kerosene are petroleum-derived products and most of them are flammable, for which reason they are commonly referred to as ignitable liquids (ILs), and they must therefore be handled carefully. PDPs have similar compositions as they have a common source, crude oil. The refining processes and the use of different additives make the products different from one another and define the final composition of each PDP. The constant development of new PDPs on the market, and the consequent diversity in the toxicity of each product, makes it necessary to develop fast and reliable analytical methods for the characterization of these products in order to discriminate them and allow the source to be identified in an effort to provide answers quickly in future accidents [6,7].

Gasoline, diesel, and paraffin spills are commonplace due to their expanding use in industrial processes and gasoline and diesel are the main fuels [8,9,10]. The toxicity of petroleum-derived products that have suffered a weathering process and the effect of these materials in nature have been studied previously [11,12,13]. However, the capability of identifying the nature of these products after a weathering process has not been studied in depth.

A rapid and appropriate characterization of a PDP can be important to evaluate its impact on the ecosystem, to accurately assess the risk, to avoid the possibility of a fire in the case of contact with high temperatures, to avoid the dangerous consumption of a PDP by animals with mortal results or to determine the effectiveness of cleaning methods amongst other factors [14,15,16].

In some cases, spills are not detected at the moment in which they occur, and during this time the spill can be subjected to different degradation processes such as microbial degradation, evaporation or weathering [17,18]. All of these phenomena can modify the chemical composition of the liquid, thus hindering the identification of the petroleum-derived product.

Weathering is one of the most common processes that affects this kind of liquid and it involves the faster evaporation of the most volatile compounds (VOCs) of a mixture when compared to non-volatile compounds. The remaining VOCs may be present at levels below the limits of detection of the analytical methods employed. Consequently, the less volatile compounds will evaporate more slowly and will therefore be present at a relatively higher ratio [19]. Weathering is closely related to variables such as temperature, pressure, light, and convection. To date, several studies have illustrated how these conditions can affect the relative distribution of the remaining chemical compounds [20,21,22]. For instance, this distribution has already been used to estimate the age of ignitable liquids and to predict the effect of these contaminants in nature [23,24].

A great number of research and review articles concern different methods for the determination of PDPs [24,25]. Most of these methods are based on gas chromatography-mass spectrometry (GC-MS) [26,27,28]. The identification of PDPs by GC-MS is usually carried out by visual inspection of the total ion chromatogram (TIC), extracted ion profile (EIP), and target compounds. The main drawback of this method is that interpretation of the results depends mainly on the experience of the analyst [29]. Furthermore, this procedure is time-consuming since it does not allow automation and it becomes more complicated when the PDP is not neat but degraded due to the occurrence of different natural phenomena.

The use of chemometric tools has expanded in recent years because they allow the extraction of useful information from complex data matrixes and they can be applied in classification and discrimination in an almost automatic procedure [30]. The main chemometric tools used in the discrimination of petroleum derivatives are principal component analysis (PCA) [31], linear discriminant analysis (LDA), and cluster analysis (CA). PARAFAC2 parallel factor analysis has been also applied to GC-MS data for the classification of weathered petroleum oils [32]. The use of the total ion spectrum (TIS) in combination with suitable chemometric tools is becoming an alternative to the use of TIC. TIS is the average mass spectrum across the chromatographic profile and it is independent of time. In several studies TIS has been successfully applied to the identification and classification of different petroleum-derived products [33].

Non-separative techniques are becoming a good alternative when it is sufficient to obtain a signal profile or fingerprint of the sample, and the identification of individual compounds is not required [34]. The electronic nose is a non-separative technique that is used as an alternative to chromatographic techniques and promising results have been obtained. Different types of Electronic Nose (eNose) have been applied as an analytical technique with different purposes [35,36,37] and the main differences between each type are the kind of sensor used.

In the study described here, an eNose based on headspace mass spectrometry was used and this technique provides a total ion mass spectrum (TIMS) without any chromatographic separation [38]. This eNose is characteristic because a quadrupole mass spectrometer is used as the detection system, with each fragment ion (*m*/*z* ratio) acting as a “sensor” and its abundance equivalent to the sensor signal. The TIMS obtained can be mathematically considered as an equivalent to the TIS and this is characteristic of each sample. The chemometric treatment of the signals obtained provides a fingerprint that can be used for the rapid identification or characterization of the petroleum-derived products.

The eNose has previously been optimized and validated for the discrimination of different neat ignitable liquids and ignitable liquid residues in fire debris [39,40,41,42]. A previous study has already demonstrated the capacity of the eNose for the discrimination of different neat PDPs poured onto different materials but without delayed sampling time [41]. However, a preliminary study on the effect of weathering by evaporation in neat gasoline samples by eNose demonstrated that the weathering process and the volume of liquids used affected the chemical fingerprint of the gasoline [43]. The results obtained demonstrated that the weathering process affected the chemical fingerprint of the gasoline. For this reason, the aim of the study described here is two-fold: (i) To evaluate the capacity of the eNose for the identification and discrimination of different weathered petroleum-derived products (gasoline, diesel, and paraffin); (ii) To study the effect of the type of supporting material (pine wood, cork, paper and cotton sheet) and the volume of PDP used in the identification of the weathered PDPs.

## 2. Materials and Methods

### 2.1. Samples

A total of 444 petroleum-derived weathered samples were created using different hydrocarbons and supporting materials:

- Three common petroleum-derived products (PDPs) were used: gasoline (RON 95), diesel (normal diesel), and paraffin (commercial ignitable liquid-Zibro Fire). All of the PDPs were purchased from local markets and gas stations.
- Four different substrates with different porosities were used as supporting materials: pine wood, natural cork, paper, and cotton sheet. These materials were chosen because they could easily be found near to the spill zones. Square pieces of substrate with a width of 0.5 cm were used.
- Volumes of 40 μL and 80 μL of PDP were chosen to study the weathering process.

Weathered samples were created as follows: Each support was placed in the middle of the base of a 10 mL open vial (Agilent Crosslab) and the appropriate volume of PDP was added. The vials were kept open (uncapped). All samples were placed inside the laboratory hood with strict control of temperature (25 °C) and the flow of the laboratory hood was zero, with the aim of simulating natural conditions for the evaporation/weathering process. At different times (0 h, 6 h, 12 h, 24 h, 72 h, 1 week, 15 days, 21 days and 1 month (30 days)) the vials with both volumes for each type of substrate and hydrocarbon were closed and analyzed. All samples were prepared in duplicate.

The samples were named using the letters G, D or P when the PDPs used were gasoline, diesel, or paraffin, respectively, and W when the support used was wood, C for cork, P for paper and S for cotton sheet. The volume and the time were noted in the name and the replicate was also recorded.

For example, the first replicate with 40 μL of gasoline on wood for 15 days was named “G_W_40μL_15d_1”.

### 2.2. Acquisition of eNose Spectra

Alpha Moss eNose (Toulouse, France) was the system chosen to analyze the weathered samples; this system consists of an HS 100 static headspace autosampler and a Kronos quadrupole mass spectrometer, used as a multiple channel sensor. The system had an autosampler oven where closed vials were placed to generate the headspace. The conditions used to generate the headspace were a temperature of 145 °C and 500 rpm of agitation for 10 min. A volume of 4.5 mL of headspace was extracted using a 5 mL gas syringe and the sample was injected into the mass spectrometer with an injection speed of 75 μL/s. The gas syringe was heated above the sample temperature (150 °C) to avoid condensation phenomena. Between each sample injection, the gas syringe was flushed with carrier gas (nitrogen) for 120 s and with a fill speed of 100 μL/s to avoid cross-contamination. These conditions were previously optimized by our research group.

The components in the headspace of the vials were passed directly to the mass detector without any chromatographic separation or sample pre-treatment and the total analysis time per sample was approximately 12 min.

The resulting total ion mass spectrum (TIMS) gives a fingerprint for each measurement, an ion electron impact spectrum recorded in the mass charge to ratio range (*m*/*z*) 45–200. Instrument control was achieved using the Residual Gas Analysis software package (RGA) and Alpha Soft 7.01 software from Alpha Moss (Toulouse, France).

### 2.3. Data Analysis and Software

TIMS data were analyzed by chemometric tools using the statistical computer package SPSS 22.0 (SPSS Inc., Armonk, NY, USA) for the Hierarchical Cluster Analysis (HCA) and Linear Discriminant Analysis (LDA).

## 3. Results and Discussion

A recent study carried out on the weathering process showed the effect of this phenomenon in the analysis of gasoline samples by eNose [42]. During this investigation, the presence of two different patterns or fingerprints were observed, one for samples from 6 h to 12 h of weathering and a second that covers samples from 24 h to 1 month of weathering. In this research it was also demonstrated that the support used did not influence the weathering process.

Based on previous results that showed how the chemical fingerprint is affected by the weathering process, the aim of this study was to evaluate the capacity of the HS-MS for the identification of the presence/absence of different PDPs (gasoline, diesel and paraffin) in unknown samples that have suffered a weathering process over a period of four weeks in order to identify any limitations of this technique.

### 3.1. Identification of Gasoline, Diesel, and Paraffin on Different Supports

The total ion mass spectrum (TIMS) in the mass charge to ratio range 40–200 *m*/*z* for each situation was used in the study. A chemometric study that included non-supervised and supervised techniques was carried out. A total of 444 weathered samples at different times (0 h, 6 h, 12 h, 24 h, 72 h, 1 week, 15 days, 21 days, and 30 days) using different PDPs and supporting materials were considered in the study. All analyses were carried out in duplicate by eNose.

Supports without any PDP were also analyzed with the aim of evaluating the possible similarities between PDP samples that had suffered a long weathering process and supports without any PDP.

Four supports materials were considered with the aim of assessing any possible influence of the porosity of the material on the development of the weathering process of hydrocarbons. The impact of the porosity of the material could require a different treatment of the data depending on the sample collected by analysts.

#### 3.1.1. Identification in Samples Without Weathering

The potential of the eNose to discriminate hydrocarbons has been demonstrated before in our group [41], however, weathered samples were not included in that study. The eNose system in combination with pattern recognition tools was able to discriminate the PDPs samples according to the type of liquid when 80 μL was added to different surfaces. The starting point in the present study was to check if there was an influence of the volume used in the discrimination of neat PPDs. For this reason, two different volumes were considered (80 μL and a reduced volume of 40 μL). In order to increment the heterogeneity of the samples an additional supporting material (paper) as well as an additional PDP (paraffin), were included. Paraffin was chosen as PDP because of it is chemically very similar to diesel, so it could be not easily discriminated from diesel.

So, samples of gasoline, diesel, and paraffin that had not suffered a weathering process (0 h) in four supports were analyzed with the aim of demonstrating the capacity of the eNose to differentiate neat hydrocarbons on different supporting materials even when different volumes are used.

**Figure 1:**
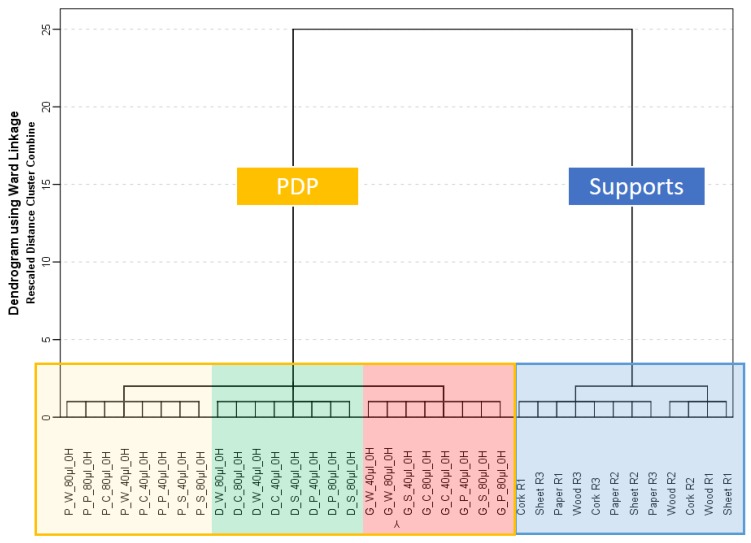
Dendrogram obtained in the hierarchical cluster analysis (HCA) using total ion mass spectrum (TIMS) for samples without weathering (*n* = 60) with and without petroleum-derived products (PDPs).

A hierarchical cluster analysis (HCA) was carried out with samples that had not suffered weathering for gasoline, diesel, and paraffin on four supports when using different volumes (40 μL and 80 μL). Supports without PDPs were also included, so a total of 60 samples were used for this analysis. Samples were normalized to the maximum and Ward’s method with Euclidian distance was used in the HCA. Average values of the intensities for replicas were used for a better visualization. The results are graphically displayed in the dendrogram shown in Figure 1. As reported previously [41], a full classification of the samples according to the presence/absence of the liquids was obtained, in addition, the support and the type of PDPs were also obtained. In addition, it was shown that when the samples were not weathered there was no influence on the volume used in this classification analysis.

It was also observed, that the eNose system was able to discriminate between paraffin and diesel samples. Based on these results, it was proceeded with the main goal of the present work, the study of the capacity of the eNose in combination with chemometric tools to discriminate the samples that have been suffered weathering process (by natural evaporation) and the study of possible influences of the volume or supporting material used during the weathering.

#### 3.1.2. Identification of Weathered PDPs

**Figure 2:**
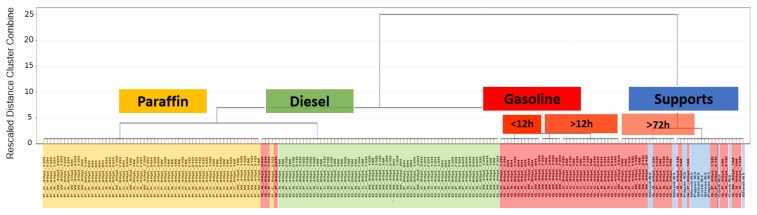
Dendrogram obtained in the HCA using average TIMS for all the weathered samples (*n* = 200) with and without PDPs.

The capacity of the eNose in the identification of weathered samples was evaluated. To do so, 40 μL and 80 μL of gasoline, diesel or paraffin were poured onto a piece of pine wood, natural cork, paper, or cotton sheet. The samples were taken at different times and analyzed by eNose. A total of 9 weathered samples were discarded as outliers. First, an HCA using all *m*/*z* intensities in the range 45–200 *m*/*z* of all the weathered PDP samples (D_387X156_) was performed. The aim of this analysis was to study the possible tendencies of the samples to be grouped according to the presence/absence and the type of PDP regardless of the weathering time or volume used. Ward’s method and Euclidean distance were again used. The results of this analysis are plotted in the dendrogram in Figure 2, where all of the samples are listed according to the level of similarity (dissimilarity). Average values were represented for a better visualization. A full classification of all the samples according to the type of PDP was not obtained. However, a tendency for grouping according to the presence or absence of PDP and also the type of PDP used was observed. As can be seen in Figure 2, two large clusters (Cluster A and B) were obtained. Cluster A is divided into three sub-clusters according to the type of PDP (paraffin, diesel, or gasoline). Besides, substrates with paraffin (yellow) and diesel (green) are linked at a shorter distance than the group with the remaining gasoline samples (read). This means that samples with paraffin and diesel are chemically more similar than samples with gasoline. Moreover, the gasoline group is further divided into small sub-clusters according to the weathering time. Lastly, Cluster B contains all of the substrates free of PDPs but also, the samples of gasoline after 15 days of weathering (violet).

The results discussed above indicate that data from the eNose used for this analysis are mainly related to those compounds responsible firstly for the discrimination of the samples according to the presence/absence of PDPs and secondly according to the type of PDPs. An influence regarding with the weathering time was also observed in the case of samples with gasoline. In fact, samples of gasoline weathered for longer than 72 h were classified together with the substrates without PDP.

As a full discrimination was not achieved, the same data matrix (D_387X156_) was used for a linear discriminant analysis (LDA). LDA is based on a supervised analysis of a number of samples with the purpose of determining equations that allow us to classify the samples in the groups previously established. In this case, five groups were considered a priori (Table 1). Samples with gasoline were divided into two different groups according to the weathering time as it was reported on a previous study [41]. The method selected for the discriminant analysis was Wilk’s lambda stepwise method leaving one out for the classification (cross validation).

A 100% of the original cases and a 94.8% of cross-validated grouped cases were correctly classified (Table 2). None of the samples with liquid was classified as a substrate alone or vice versa. All the classification errors (*n* = 20) corresponded to gasoline samples. However, 11 of those 20 samples were gasoline samples after 24 h of weathering that were misclassified in the group of gasoline samples but before 24 h of weathering. This is probably due to the volume used. If a higher volume is used the weathering is delayed. 4 gasoline samples from the group 6–12 h of weathering were misclassified as diesel samples and 5 as paraffin samples. Based on this, it can be considered that only a 2.3% of the samples were erroneously classified according to the type of PDP used.

**Figure 3:**
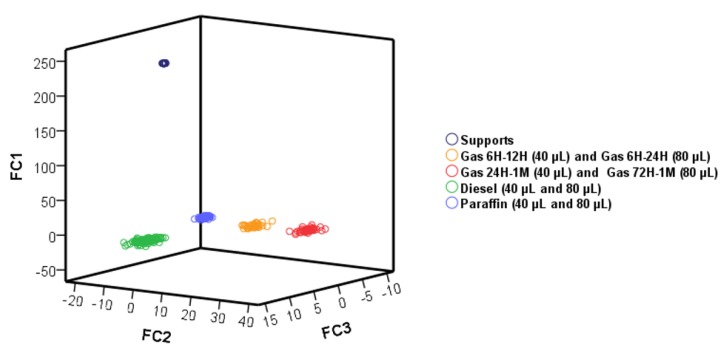
Score plot for all the samples based on the three discriminant functions from linear discriminant analysis (LDA).

Four canonical discriminant functions were obtained; the score values regarding three of them were represented (Figure 3), showing a full discrimination of the samples according to the presence/absence and type of PDP.

**Figure 4:**
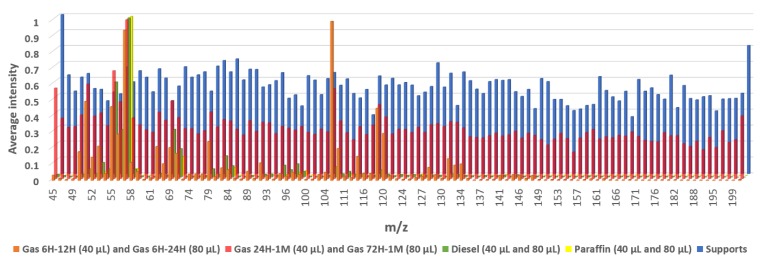
Average intensities of the *m*/*z* of the classification function coefficients.

With the purpose of obtaining the characteristic profile of each petroleum-based product, the *m*/*z* values selected as being important in the Fisher’s linear discriminant function during the LDA were represented (Figure 4). The average *m*/*z* values for each group were represented and normalized to the base peak at 100%. It can clearly be seen the differences in the intensity values of the selected *m*/*z* from samples with and without PDPs. Substrates free of PDPs gave similar intensities for all *m*/*z* and did not show a distinctive signal. However, samples that contained PDPs presented a few characteristic signals. In samples with diesel or paraffin the highest signal was *m*/*z* 57, but the intensities and the ratios of the rest of the signals were different. In samples with gasoline weathered for not longer than 24 h, the maximum signal was *m*/*z* 105, although *m*/*z* 57 presented high intensity too. The situation changed in the case of samples with gasoline after 24 h of weathering, since *m*/*z* 57 become the highest while the intensity of the *m*/*z* 105 decreased.

The results indicate that eNose combined with suitable chemometrics is capable of determining not only the presence of petroleum-derived products, but also their nature even after 1 month of weathering. Chemometrics identify the *m*/*z* values that are decisive in the discrimination of gasoline, diesel, and paraffin to give the characteristic profile of each sample. Therefore, the phenomenon of weathering under the studied conditions is not a limitation in the detection and discrimination of hydrocarbons by the eNose.

## 4. Conclusions

The capacity of the eNose technique as a tool to detect and determine the presence of different petroleum-derived products in an unknown sample, regardless of the weathering process, has been demonstrated. The weathering process is not a disadvantage in the determination and identification of a petroleum-derived product and this allows analysts to collect and analyze samples up to one month after a spillage.

Furthermore, eNose combined with chemometrics allows the determination of the *m*/*z* values required for the identification of PDPs, which in turn provides a method to identify the presence of PDPs in unknown samples within minutes. 97.7% of the samples were correctly classified according to the type of PDP used. It should be noted that none of the samples with PDP were classified as support free of PDP and vice versa.

The support or the volume used did not influence on the discrimination of the samples. Only influences in the amount of gasoline used were observed since when a higher volume was used, the weathering process was delayed.

The eNose has several advantages since it is a green technique and it is also fast and easy to use in routine analysis. These characteristics mean that this method could be used to identify the presence of spills and remove the danger for a very low cost.

## Figures and Tables

**Table 1:** Groups stablished a priori for the LDA.

Group Code	Samples
0	Supports
1	Gas 6H-12H (40 μL) and Gas 6H-24H (80 μL)
2	Gas 24H-1M (40 μL) and Gas 72H-1M (80 μL)
3	Diesel (40 μL and 80 μL)
4	Paraffin (40 μL and 80 μL)

**Table 2:** Classification results from the linear discriminant analysis (*n* = 387).

		GR	Predicted Group Membership					Total
			Supports	Gas 6H-12H	Gas 24H-1M	Dies	Par	
Original ^a^	Count	0	12.0	0	0	0	0	12.0
		1	0	37.0	0	0	0	37.0
		2	0	0	87.0	0	0	87.0
		3	0	0	0	124.0	0	124.0
		4	0	0	0	0	127.0	127.0
	%	0	100.0	0	0	0	0	100.0
		1	0	100.0	0	0	0	100.0
		2	0	0	100.0	0	0	100.0
		3	0	0	0	100.0	0	100.0
		4	0	0	0	0	100.0	100.0
Cross-validated ^b^	Count	0	12.0	0	0	0	0	12.0
		1	0	37.0	0	0	0	37.0
		2	0	11.0	67.0	4.0	5.0	87.0
		3	0	0	0	124.0	0	124.0
		4	0	0	0	0	127.0	127.0
	%	0	100.0	0	0	0	0	100.0
		1	0	100.0	0	0	0	100.0
		2	0	12.6	77.0	4.6	5.7	100.0
		3	0	0	0	100.0	0	100.0
		4	0	0	0	0	100.0	100.0

^a^ 100.0% of original grouped cases correctly classified; ^b^ Cross validation is done only for those cases in the analysis. 94.8% of cross-validated grouped cases correctly classified.
